# The Psychometric Properties of the Persian Migraine-Specific Quality of Life Questionnaire Version 2.1 in Episodic and Chronic Migraines

**DOI:** 10.1155/2013/950245

**Published:** 2013-08-27

**Authors:** Alireza Zandifar, Samaneh Sadat Masjedi, Faraidoon Haghdoost, Fatemeh Asgari, Navid Manouchehri, Mahboobeh Banihashemi, Mohammad Reza Najafi, Abbas Ghorbani, Behzad Zolfaghari, Ali Gholamrezaei, Vahid Shaygannejad, Mohammad Saadatnia

**Affiliations:** ^1^Department of Physiology, Physiology Research Center, Isfahan University of Medical Sciences, Isfahan 81745-319, Iran; ^2^Medical Student Research Center, Isfahan University of Medical Sciences, Isfahan 81745-319, Iran; ^3^Department of Neurology and Isfahan Neurosciences Research Center, Isfahan University of Medical Sciences, Isfahan 81745-319, Iran; ^4^Department of Pharmacognosy and Isfahan Pharmaceutical Sciences Research Center, School of Pharmacy, Isfahan University of Medical Sciences, Isfahan 81745-319, Iran; ^5^Poursina Hakim Research Institution, Isfahan, Iran

## Abstract

*Background*. Migraine-specific quality of life (MSQ) is a valid and reliable questionnaire. Linguistic validation of Persian MSQ questionnaire, analysis of psychometric properties between chronic and episodic migraine patients, and capability of MSQ to differentiate between chronic and episodic migraines were the aims of this study. *Method*. Participants were selected from four different neurology clinics that were diagnosed as chronic or episodic migraine patients. Baseline data included information from MSQ v. 2.1, MIGSEV, SF-36, and symptoms questionnaire. At the third week from the baseline, participants filled out MSQ and MIGSEV. Internal consistency (Cronbach alpha) and test-retest reproducibility (intraclass correlation coefficients) were used to assess reliability. Convergent and discriminant validities were also assessed. *Results*. A total of 106 participants were enrolled. Internal consistencies of MSQ among all patients, chronic and episodic migraines, were 0.92, 0.91, and 0.92, respectively. Test-retest correlation of MSQ dimensions between visits 1 and 2 varied from 0.41 to 0.50. Convergent, item discriminant, and discriminant validities were approved. In all visits MSQ scores were lower in chronic migraine than episodic migraine; however, the difference was not statistically significant. *Conclusion*. Persian translation of MSQ is consistent with original version of MSQ in terms of psychometric properties in both chronic and episodic migraine patients.

## 1. Introduction

Quality of life (QOL) is a key point in assessing burden of disease and is highly useful in estimating the effectiveness of interventions in clinical trials and routine clinical treatment efficacy evaluations [1, 2]. Instruments that measure QOL consist of generic and disease-specific tools, both of which can be used to evaluate health-related quality of life (HRQOL). Although generic tools such as SF-36 and SF-12 questionnaires are valuable yet they cannot measure individual burden of any particular disease and are less sensitive to relevant changes in HRQOL domains within the context of specific disease. As a result there is a need for specific tools for each disease such as diabetes, arthritis, inflammatory bowel disease, and migraine [3, 4].

Migraine is a chronic disease that is associated with morbidity and significantly impaired QOL in patients [5–7]. Migraine is highly prevalent in productive years and causes noticeable amounts of direct and indirect expenses [8, 9]. Measuring QOL through assessment of frequency and severity of attacks cannot establish the exact effect of migraine on HRQOL entirely [10]. Determination of QOL domains through specific instruments has been developed. Recent specific instruments include MSQOL (Migraine-specific Quality of Life Measure) [11], 24 h MQoLQ (24-hourMigraine Quality of Life Questionnaire) [12] and MSQ (Migraine-Specific Quality of Life Questionnaire) [13, 14].

MSQOL is an instrument for assessment of migraine-specific quality of life for unspecific long period of time [11] and 24 h MQoLQ can be used only in 24 hours after treatment that is a very short term migraine-specific quality of life questionnaire [12].

MSQ is a well known instrument that has been widely used in researches and clinical practice that measures QOL over the past 4 weeks across 3 dimensions; role restrictive (RR), role preventive (RP), and emotional function (EF) [13, 15]. MSQ v. 2.1 consists of 14 questions, seven questions in RR dimension, three questions in PR dimension and four questions in EF dimension. Analysis of MSQ v. 2.1 has shown high internal consistency, moderate convergent validity and strong reliability [14]. Each question of MSQ v. 2.1 has six available answers; none of the time, a little bit of the time, some of the time, a good bit of the time, Most of the time and all of the time that has been scored between zero and 100, respectively. Higher scores in MSQ indicate better QOL state [16–18].

To date there have been no valid and reliable translations of any migraine questionnaires to assess the quality of life in Iranian patients. The aim of this study was to evaluate MSQ reproducibility factors and test its validity and reliability in Iranian patients after using a standard forward-backward translation process. We also analyzed the psychometric properties of this questionnaire between chronic and episodic migraine patients. The capability of MSQ to differentiate between chronic and episodic migraine was also assessed.

## 2. Methods and Materials

### 2.1. Patient and Setting

Subjects were known cases of migraine, selected from regular patients at four neurology clinics in Isfahan, Iran, during their usual clinical follow-ups. Patients were diagnosed with migraine by a neurologist. All subjects were under treatment by at least one migraine prophylactic drug and one drug, used as an analgesic agent during the headache episodes. The patients' condition was stable, and the stability was defined based on neurologists' opinion as no need of any modification in drug type or dosage. The migraine was diagnosed based on International Headache Society (IHS) criteria [19]. All patients were divided into two groups according to the frequency of headache in one month. Episodic migraine (EM) was defined as a number of headache less than 15 days per month for at least three months, and the patient with frequency of headache more than 15 days per month for at least three months was classified as chronic migraine (CM) [19]. Patients participated in the study for a three-week period and were asked to complete the MSQ v. 2.1 questionnaire three times during the study in the first day (visit 1) and 3rd week (visit 2) after enrollment. The sociodemographic and headache characteristics of all the subjects including age, sex, marital status, living place, education, frequency and duration of headaches, and aura were asked in the first visit (baseline). SF-36 questionnaire was administered in the first visit. Patients completed MIGSEV scale on each visit. 

### 2.2. Assessment

MIGSEV scale, developed by EL Hasnaoui et al. in 2003, is a simple severity scale with four items including intensity of pain, disability in daily activity, tolerability of headaches, and nausea that categorize patients in three groups of intensity, mild, moderate, and severe. This instrument is highly reliable, reproducible, and sensitive [20, 21].

SF-36 is a generic health survey that consists of two components of mental and physical wellbeing. Physical component is assessed through four dimensions: physical functioning (10 questions), role-physical (4 questions), bodily pain (2 questions), and general health (5 questions). Mental component is assessed through the other four dimensions: vitality (4 questions), social functioning (2 questions), role-emotional (3 questions), and mental health (5 questions). Each dimension and each component score from zero to 100. Higher scores stand for better quality of life. SF-36 questionnaire has been already translated into more than a hundred different languages. The validity and reliability of Persian version of SF-36 have been proven by Montazeri et al. in 2005 [22].

### 2.3. Linguistic Validation

Permission for translation was given by GlaxoSmithKline Inc. the owner of MSQ v. 2.1 copy right. The multistage linguistic validation was based on standard forward-backward methodology. A panel of two neurologists (M Saadatnia and V Shaygannejad), one translator and a linguistics expert (A Okhovat), evaluated the first draft of the translation, and results were reported to the developers, and their comments were used to edit the Persian version. It was then used in a pilot study with 50 patients and five neurologists for “cognitive debriefing” and “clinician's review.” Final Persian version of MSQ v. 2.1 was prepared according to the results of pilot study and comments of questionnaire developers [23].

### 2.4. Psychometric Properties

Internal consistency and test-retest reliability of MSQ questionnaire were assessed by Cronbach alpha and intraclass correlation coefficients (ICC), respectively. Both Cronbach alpha and ICC were calculated for all participants and also EM and CM patients separately. Cronbach alpha was calculated for each visit separately. The provided data was used to assess the correlation between individual matching questions in the first and second visits. 

Convergent validity of MSQ was assessed through measurement of Pearson correlation coefficient between MSQ scores (total score and each dimension of MSQ) and two components of SF-36 (mental and physical) scores. 

Analysis of convergent validity was done only in the first visit both among the all participants and subtypes of migraine (EM and CM) separately. Higher scores of MSQ questionnaire were anticipated to be associated with higher scores of SF-36.

Discriminant validity was assessed through comparison between different grades of MIGSEV scale in each dimension and in total MSQ scores. We expected a significant difference in each dimension and in total MSQ scores between different grades of MIGSEV scale. 

Correlation between each question and its related dimension and also correlation between each question and the total score of MSQ were calculated to measure the item discriminant validity of MSQ.

Total scores of MSQ were compared between EM and CM patients. We expected significant differences between EM and CM patients.

### 2.5. Data Analysis

Cronbach alpha was used to measure internal consistency. *α* ≥ 0.7 and ≥0.8 was considered the acceptable and excellent internal consistency, respectively. 

ICC was used to examine test-retest reproducibility, and Pearson correlation coefficient was used in convergent, item discriminant validity calculations. The ICCs and Pearson correlation coefficient (*r*) were reported. We assumed ICCs or *r* < 0.3 as weak correlation, 0.3 < ICCs or *r* < 0.6 as moderately, and ICCs or *r* > 0.6 as highly correlated. ANOVA was used to compare the mean scores between groups.

## 3. Results

### 3.1. Patients' Demographics

A total of 106 patients (20.8% males, mean age (±SD) = 30.20 ± 8.81 years) were enrolled in the study. In the first and second visits, 105 and 83 of enrolled patients had participated, respectively. Sociodemographic and clinical characteristics of the patients are summarized in Tables 1 and 2. Based on the classification criteria defined in the method section, 73.2% (*n* = 74) had episodic migraine and 26.8% (*n* = 27) had chronic migraine. 

### 3.2. Reliability

Cronbach alpha for MSQ questionnaire were 0.92 and 0.95 in the first and second visits, respectively. For CM and EM patients, the internal consistency analysis showed Cronbach alpha at 0.91 and 0.92, respectively. Also the internal consistency of the questionnaire according to the three dimensions (RR, RP, and EF) in the first visit was analyzed (Cronbach *α* = 0.84, 0.87, and 0.79 for RR, RP, and EF dimensions, resp.). 

Test-retest reliability was tested for all of the patients enrolled in this study at visit 2 comparing with visit 1. The mean total MSQ scores in first and second visits were 45.92 ± 2.12 (median: 46.11) and 52.4 ± 2.46 (median: 54.20), respectively (ICCs = 0.49, *P* value <0.001). The test-retest reproducibility analysis of MSQ for the three dimensions of MSQ is also reported in Table 3.

### 3.3. Validity

For the first visit, the correlations of each question with total MSQ score are shown in Table 4. All the questions were significantly correlated with total MSQ score (*r* = 0.44–0.81, *P* value <0.001) (Table 4).

As Table 5 presents, comparison of the mean MSQ scores between the three grades of MIGSEV showed significant differences (*P* < 0.001). 

The total MSQ score in the first visit was correlated with SF-36 mental and physical scores (0.41 and 0.46, resp., *P* < 0.001). Also there was a significant correlation between scores of SF-36 components (physical and mental) and the total scores of MSQ in EM patients (*r* = 0.47 and 0.43, *P* < 0.001). In CM patients a significant correlation was also found between scores of SF-36 physical component and total MSQ scores (*r* = 0.42, *P* < 0.05); however, no significant correlation was found between scores of SF-36 mental scores and total MSQ scores (*r* = 0.27).  Table 6 reports the correlation of all MSQ dimensions with SF-36 mental and physical scores. 

### 3.4. Discrimination between EM and CM by MSQ

Comparison of EM and CM according to the total MSQ score showed that there were no significant differences between the two types of migraine in the mean score of MSQ questionnaire in the first visit (mean ± SE = 47.15 ± 2.37 and 44.00 ± 3.62 for EM and CM, resp., *P* = 0.245) and also second visit (mean ± SE = 53.08 ± 2.94 and 47.69 ± 4.67 for EM and CM, resp., *P* = 0.180). 

## 4. Discussion

This study stated the proper validity and reliability of Persian translation of MSQ and that it can be used in clinical assessments in both EM and CM patients. 

### 4.1. Reliability

The results revealed a high level of internal consistency (Cronbach *α* in the first and second visits was 0.92 and 0.95). Also remarkable internal consistencies were reported for each dimension (Cronbach *α* = 0.84, 0.87, and 0.79 for RR, RP, and EF dimensions, resp.). Our findings match the results of prior studies, and EF dimension had the lowest Cronbach *α* since it had the least number of questions [13, 14, 24]. Similar to the previous study about MSQ validation for EM and CM patients, we found high internal consistency for both EM and CM patients (*α* > 0.9) [25].

Intraclass correlation coefficients indicated an acceptable test-retest reliability between consequent visits in all dimensions and total scores (ICCs > 0.4). Lower correlations in EF dimension were expected considering the variable emotional parameter of this dimension. Our data is consistent with original MSQ v. 2.1 validation study [14].

### 4.2. Validity

As we anticipated correlations were found between MSQ total score and SF-36 components (physical and mental). MSQ dimensions were also related to SF-36 components. Higher correlation levels were observed between MSQ scores and physical component of SF-36; even EF dimension of MSQ was more related to physical component of SF-36 which is in contrast with the results from prior studies [14, 26]. Also results revealed stronger correlation between MSQ and SF-36 physical than SF-36 mental component in both EM and CM patients. This might indicate that physical impairment has a relatively higher contribution to a decreased quality of life in migraine patients. Lipton et al. reported a similar result that physical and mental components of SF-36 were lower in migraine patients with moderate to high disability versus healthy controls, but only the difference in physical component was statistically significant [27]. 

The insufficient levels of correlation between MSQ and SF-36 in our study were similar to results of original validation study of MSQ v. 2.1 [14]. However, SF-36 is a general instrument for assessing quality of life and cannot completely reflect the quality of life in migraine patients. We speculate that this might be responsible for relatively moderate levels of correlation between MSQ and SF-36 [14]. 

Similar to our results, previous studies reported a moderate correlation between MSQ and migraine symptoms of patients, severity and frequency of headaches, and other tools that measure severity such as HIT-6 [13, 14, 25]. This can be attributed to the fact that severity of migraine symptoms is not highly related to quality of life [28]. 

We compared the MSQ total scores between different MIGSEV grades and found a significant difference. This implies that MSQ is able to discriminate different severities of migraine in different patients.

Scores from each MSQ question had a high correlation with the MSQ total score. There was also a higher correlation between scores from each question with its dimension than with the total MSQ score. Item-total correlation and relatively higher intradimensional correlations were previously reported in the literature. Our results are consistent with MSQ v. 2.1 original study [14]. On the contrary we did not find the low relation between items 10, 11, and 12 with total scores as it was mentioned by similar studies [13, 24, 26]. 

### 4.3. EM and CM Discrimination by MSQ

Significant difference was not found in MSQ scores between EM and CM patients comparing first and second visits. Definitions of EM and CM are based on frequency features of the migraine [19]. Wang et al. showed that correlation between QOL and frequency of headache is not strong, therefore, barely accounted for any clinical significance [29]. As quality of life is not only affected by the frequency of headache and there are several parameters affecting the QOL in migraine patients, MSQ might fail to successfully differentiate EM from CM; nevertheless Bagley et al. reported in their study that MSQ can discriminate EM from CM [25]. Also our study had a smaller sample size which could explain the lack of significance. 

To our knowledge this is the first study to evaluate reliability of MSQ questionnaire in patients with chronic migraine through test-retest reliability method [25]. Although our small sample size in CM group is most important limitation to confirm this result, we used a specialist opinion in diagnosing our participants with migraine [25]; however, we selected them from referred patients to a specialty clinic which might narrow our sample population to patients with relatively more severe migraine. Due to this limitation we suggest that further studies should be conducted among a larger sample of migraine patients that also include patients who are not referred to specialists.

This is the first linguistic validation of MSQ questionnaire in Persian. We used a formal translation process through standard methods and demonstrated that MSQ had proper psychometric properties and was adequately reliable and valid for Iranian migraine patients. We also concluded that MSQ can relatively differentiate between EM and CM. 

## Figures and Tables

**Table 1:** Demographic characteristics of the patients.

	*n*	%
Sex		
Male	22	20.8
Female	84	29.2
Marital status		
Single	31	30.4
Married	71	69.6
living place		
Urban	59	64.8
Rural	32	35.2
Education		
Primary school	2	1.9
Guidance school	18	17.1
Diploma	49	46.7
Bachelor and above	36	34.3

**Table 2:** Clinical characteristics of the patients.

	*N*	%
Frequency of headache		
Every day	10	9.4
More than once a week	56	52.8
Once a week	14	13.3
Less than once a week	26	24.5
Duration of headache		
≤3 hours	77	81
3–24 hours	18	19
>24 hours	0	0
Aura		
Yes	34	32.4
No	71	67.6

**Table 3:** The intraclass correlation coefficients between visits 1 and 2.

	Visit 1 all patients	Visit 2 all patients	ICCs
RR dimension	43.03 ± 2.00	49.18 ± 2.37	0.50**
RP dimension	53.23 ± 2.52	57.31 ± 2.70	0.49**
EF dimension	43.25 ± 2.63	59.40 ± 2.83	0.41**
Total MSQ score	45.92 ± 2.12	52.4 ± 2.46	0.49**

Q: question; RR: role restrictive; RP: role preventive; EF: emotional functioning; ICCs: intraclass correlation coefficients; *P < 0.01; **P < 0.001; each value is mean ± SE.

**Table 4:** Correlation of each question of MSQ questionnaire with total score and its dimension.

	MSQ questionnaire total score and dimensions	
	Total MSQ score	Related dimension
Q1	0.44*	0.58*
Q2	0.48*	0.69*
Q3	0.72*	0.79*
Q4	0.65*	0.77*
Q5	0.70*	0.75*
Q6	0.76*	0.77*
Q7	0.58*	0.63*
Q8	0.78*	0.87*
Q9	0.81*	0.87*
Q10	0.69*	0.85*
Q11	0.75*	0.79*
Q12	0.72*	0.78*
Q13	0.76*	0.89*
Q14	0.73*	0.84*

Q: question; RR: role restrictive; RP: role preventive; EF: emotional functioning; data are given as Pearson correlation coefficient; *P < 0.001.

**Table 5:** Comparison of the mean MSQ scores between three grades of MIGSEV scale.

	MIGSEV grades			
	MIGSEV grade 1 (*n* = 31)	MIGSEV grade 2 (*n* = 27)	MIGSEV grade 3 (*n* = 44)	*P* value
RR dimension	51.23 ± 3.65	43.70 ± 2.75	33.40 ± 2.45	<0.001
RP dimension	65.48 ± 3.45	55.55 ± 3.83	43.75 ± 3.38	<0.001
EF dimension	53.97 ± 4.62	46.66 ± 4.74	31.66 ± 3.04	<0.001
MSQ total	56.61 ± 3.66	48.64 ± 3.29	36.19 ± 2.49	<0.001

RR: role restrictive; RP: role preventive; EF: emotional functioning; each value is mean ± SE, *n*: number; *P < 0.001; each value is mean ± SE.

**Table 6:** Correlation of SF-36 total, mental and physical scores with MSQ scores.

	Total patients (*n* = 101)		EM (*n* = 71)		CM (*n* = 26)	
	SF-36 physical	SF-36 mental	SF-36 physical	SF-36 mental	SF-36 physical	SF-36 mental
Role restrictive	0.37**	0.34**	0.39**	0.33**	0.29	0.20
Role preventive	0.47**	0.42**	0.54**	0.49**	0.29	0.23
Emotional function	0.39**	0.35**	0.33**	0.33**	0.53**	0.29
Total MSQ	0.46**	0.41**	0.47**	0.43**	0.43*	0.27

*n*: number; CM: chronic migraine; EM: episodic migraine; *r*: pearson correlation coefficient; *P < 0.05; **P < 0.001; each value is mean ± SE.

## References

[1] Sanders C, Egger M, Donovan J, Tallon D, Frankel S (1998). Reporting on quality of life in randomised controlled trials: bibliographic study. *British Medical Journal*.

[2] Calvert MJ, Freemantle N (2003). Use of health-related quality of life in prescribing research. Part 1: why evaluate health-related quality of life?. *Journal of Clinical Pharmacy and Therapeutics*.

[3] Guyatt GH, Bombardier C, Tugwell PX (1986). Measuring disease-specific quality of life in clinical trials. *Canadian Medical Association Journal*.

[4] Duru G, Auray J-P, Gaudin A-F (2004). Impact of readache on quality of life in a general population survey in France (GRIM2000 study). *Headache*.

[5] Pompili M, Serafini G, di Cosimo D (2010). Psychiatric comorbidity and suicide risk in patients with chronic migraine. *Neuropsychiatric Disease and Treatment*.

[6] Serafini G, Pompili M, Innamorati M (2012). Gene variants with suicidal risk in a sample of subjects with chronic migraine and affective temperamental dysregulation. *European Review for Medical and Pharmacological Sciences*.

[7] Pompili M, Serafini G, Innamorati M (2010). Patient outcome in migraine prophylaxis: the role of psychopharmacological agents. *Patient Related Outcome Measures*.

[8] Lipton RB, Stewart WF, Diamond S, Diamond ML, Reed M (2001). Prevalence and burden of migraine in the United States: data from the American Migraine Study II. *Headache*.

[9] Lipton RB, Stewart WF, Scher AI (2001). Epidemiology and economic impact of migraine. *Current Medical Research and Opinion*.

[10] Holroyd KA (2002). Assessment and psychological management of recurrent headache disorders. *Journal of Consulting and Clinical Psychology*.

[11] Wagner TH, Patrick DL, Galer BS, Berzon RA (1996). A new instrument to assess the long-term quality of life effects from migraine: development and psychometric testing of the MSQOL. *Headache*.

[12] Hartmaier SL, Santanello NC, Epstein RS, Silberstein SD (1995). Development of a brief 24-hour migraine-specific quality of life questionnaire. *Headache*.

[13] Jhingran P, Osterhaus JT, Miller DW, Lee JT, Kirchdoerfer L (1998). Development and validation of the migraine-specific quality of life questionnaire. *Headache*.

[14] Martin BC, Pathak DS, Sharfman MI (2000). Validity and reliability of the migraine-specific quality of life questionnaire (MSQ Version 2.1). *Headache*.

[15] Jhingran P, Davis SM, LaVange LM, Miller DW, Helms RW (1998). MSQ: migraine-specific quality-of-life questionnaire: further investigation of the factor structure. *PharmacoEconomics*.

[16] Wang S-J, Wang P-J, Fuh J-L, Peng K-P, Ng K (2013). Comparisons of disability, quality of life, and resource use between chronic and episodic migraineurs: a clinic-based study in Taiwan. *Cephalalgia*.

[17] Buse DC, Silberstein SD, Manack AN, Papapetropoulos S, Lipton RB (2012). Psychiatric comorbidities of episodic and chronic migraine. *Journal of Neurology*.

[18] Coppola G, Schoenen J (2012). Management of acute and chronic migraine.. *Current Opinion in Supportive and Palliative Care*.

[19] Headache Classification Subcommittee of the International Headache Society (2004). The International Classification of Headache Disorders: 2nd edition. *Cephalalgia*.

[20] El Hasnaoui A, Vray M, Richard A, Nachit-Ouinekh F, Boureau F (2003). Assessing the severity of migraine: development of the MIGSEV scale. *Headache*.

[21] El Hasnaoui A, Vray M, Blin P (2004). Assessment of migraine severity using the MIGSEV scale: relationship to migraine features and quality of life. *Cephalalgia*.

[22] Montazeri A, Goshtasebi A, Vahdaninia M, Gandek B (2005). The Short Form Health Survey (SF-36): translation and validation study of the Iranian version. *Quality of Life Research*.

[23] Ohbu S, Igarashi H, Okayasu H (2004). Development and testing of the Japanese version of the migraine-specific quality of life instrument. *Quality of Life Research*.

[24] Rendas-Baum R, Bloudek LM, Maglinte GA, Varon SF (2012). The psychometric properties of the migraine-specific quality of life questionnaire version 2.1 (MSQ) in chronic migraine patients. *Quality of Life Research*.

[25] Bagley CL, Rendas-Baum R, Maglinte GA (2012). Validating migraine-specific quality of life questionnaire v2.1 in episodic and chronic migraine. *Headache*.

[26] Cole JC, Lin P, Rupnow MFT (2007). Validation of the migraine-specific quality of life questionnaire version 2.1 (MSQ v. 2.1) for patients undergoing prophylactic migraine treatment. *Quality of Life Research*.

[27] Lipton RB, Liberman JN, Kolodner KB, Bigal ME, Dowson A, Stewart WF (2003). Migraine headache disability and health-related quality-of-life: a population-based case-control study from England. *Cephalalgia*.

[28] Wilson IB, Cleary PD (1995). Linking clinical variables with health-related quality of life: a conceptual model of patient outcomes. *Journal of the American Medical Association*.

[29] Wang S-J, Fuh J-L, Lu S-R, Juang K-D (2001). Quality of life differs among headache diagnoses: analysis of SF-36 survey in 901 headache patients. *Pain*.

